# In Vitro Activity of (−)-Myrtenol on Adherence, Morphogenesis and Lipase Activity in *Candida albicans* Isolated from the Oral Cavity

**DOI:** 10.3390/jof12050325

**Published:** 2026-04-29

**Authors:** Camila Mendes Soares, Larissa Alves da Silva, Luanna de Oliveira e Lima, Meryellem Bezerra Soares, Raimundo Euzebio da Costa Neto, José Maria Barbosa Filho, Felipe Queiroga Sarmento Guerra, Guilherme Maranhão Chaves, Walicyranison Plínio da Silva-Rocha

**Affiliations:** 1 Clinical Mycology Laboratory, Department of Pharmaceutical Sciences, Federal University of Paraíba, João Pessoa 58051-900, Paraíba, Brazil; camilamendes2314@gmail.com (C.M.S.); larissa.silva@academico.ufpb.br (L.A.d.S.); luanna@ltf.ufpb.br (L.d.O.e.L.); meryellem.soares@academico.ufpb.br (M.B.S.); raimundo.neto@academico.ufpb.br (R.E.d.C.N.); fqsg@academico.ufpb.br (F.Q.S.G.); 2 Pharmaceutical Technology Laboratory, Department of Pharmaceutical Sciences, Federal University of Paraíba, João Pessoa 58051-900, Paraíba, Brazil; jbarbosa@ltf.ufpb.br; 3 Laboratory of Medical Mycology, DMIC—Department of Mycology—BC, Federal University of Pernambuco, Recife 50670-901, Pernambuco, Brazil; guilherme.chaves@ufpe.br

**Keywords:** *Candida albicans*, opportunistic infections, pathogenicity, phytochemicals, myrtenol

## Abstract

*Candida albicans* is a yeast found in the oral cavity, gastrointestinal tract, and vaginal mucosa. This species is the most prevalent and virulent in conditions such as oral candidiasis. Myrtenol is a bicyclic monoterpene alcohol recognized for its antioxidant and anti-inflammatory attributes. Its primary source is the essential oil extracted from plants of the Myrtaceae family. This study evaluated the effect of (−)-myrtenol on the virulence factors of *Candida albicans*. Ten clinical isolates of *Candida albicans* and one reference strain (ATCC 90028) were used in this study. The virulence factors examined included adhesion, morphogenesis, and lipase production. Assays were conducted in the presence and absence of (−)-myrtenol, using a concentration corresponding to the minimum inhibitory concentration (MIC; 256 µg/mL). Results: The compound reduced the adherence of *C. albicans* to human oral epithelial cells (92.24 vs. 28.69), and reduced filamentation in liquid (3.17 vs. 2.57) and solid media. Furthermore, (−)-myrtenol inhibited lipase activity (0.68 vs. 1.00). Virulence factors expressed by *C. albicans* contribute to increased infection rates and, consequently, increased morbidity and mortality. The present findings demonstrate that (−)-myrtenol affects virulence-associated phenotypes of *C. albicans* in vitro. This compound represents a promising candidate for further investigation, particularly in studies addressing its mechanisms of action, safety, and potential applicability.

## 1. Introduction

The oral cavity is an environment inhabited by a variety of microorganisms, including viruses, fungi, protozoa, and bacteria, which colonize structures such as teeth, tongue, gingival sulcus, mucous membranes, and oropharynx [1,2]. In healthy individuals, yeasts of the genus *Candida* can colonize without causing harm to the host. However, under favorable conditions, these fungi can proliferate and invade adjacent tissues, leading to oral candidiasis, an opportunistic fungal infection [3].

Among the species of the genus *Candida*, *C. albicans* is considered the most prevalent, being isolated in more than 80% of cases [4]. However, other species such as *C. tropicalis*, *C. parapsilosis Complex*, *Nakaseomyces glabratus* and *Pichia kudriavzevii* can also be identified [3,5,6]. Systemic factors related to immunosuppression, malignant hematological diseases, prolonged use of antibiotics and corticosteroids, as well as local factors such as loss of vertical occlusion dimension in edentulous or partially edentulous patients, hyposalivation, poorly maintained dental prostheses, and epithelial dysplasias can contribute to the development of oral candidiasis [6,7,8].

Clinically, oral candidiasis can present in various forms, with pseudomembranous candidiasis being the most recognized [9]. Patients may have asymptomatic cases in most cases or present with pain, taste alterations, itching, and dysphagia, in addition to aesthetic issues that significantly affect quality of life [10]. Other factors to consider in the onset and progression of this disease, including its more severe forms such as candidemia, are the expression of virulence factors present in the strain, in addition to the host’s immunocompromised state.

Adhesion mechanisms play a crucial and primitive role in the onset of infections in the oral cavity, whether in the mucosa, teeth or dental materials [11]. In this context, *C. albicans* is one of the species that most effectively expresses the ability to adhere to biotic and abiotic surfaces, contributing to their greater virulence. This ability is closely related to the interaction between host cells and adhesion proteins expressed by the fungus, known as adhesins [12].

*Candida albicans* also exhibits polymorphism, meaning it can display three distinct morphologies: blastoconidia, true hyphae, and pseudohyphae [13]. This attribute, known as morphogenesis, facilitates tissue invasion by the fungus, promoting greater local damage and, depending on the patient’s condition, potentially favoring dissemination through the bloodstream [12]. Additionally, the secretion of hydrolytic enzymes, such as lipases, increase the fungus’s invasive capacity [14,15].

Despite advances in the pharmaceutical industry in developing new medications for various systemic disorders, treatment for candidiasis remains limited to the availability of the main classes of antifungals: azoles, echinocandins, and polyenes.

A systematic review with meta-analysis on antifungal resistance in *Candida albicans* isolated from the human oral cavity demonstrated that most currently available medications for the treatment of oral candidiasis remain effective. However, the study also showed that when strains were associated with patients with different comorbidities, a high rate of resistance to the antifungals used was observed [16]. This scenario of increasing resistance reinforces the need to develop new active compounds, such as natural products and their derivatives, to expand the therapeutic arsenal.

Scientific research on new therapeutic agents derived from natural products has gained increasing momentum in recent years [17]. Among the numerous compounds identified and analyzed across various studies, myrtenol (C_10_H_16_O), a monoterpene primarily found in the species *Myrtus communis* (commonly known as “true myrtle” or “common myrtle”), is well-documented in the literature for its anti-inflammatory, antioxidant, wound healing, gastroprotective, and antinociceptive properties [18,19,20].

Myrtenol can exist in the form of enantiomers such as (−)-myrtenol and (+)-myrtenol which are distinguished by their chirality. Although there are no records in the literature on the distinct biological activities between these enantiomers, Mahmoud et al. [21] using both optical isomers in an in vitro study, showed that (+)-myrtenol increased antimicrobial activity through a synergistic effect with drugs such as amikacin, fluconazole, and benzalkonium chloride in clinical isolates of *Staphylococcus aureus* and *C. albicans*. (−)-Myrtenol, on the other hand, acted in conjunction with amikacin and fluconazole in reducing biofilm formation in half of the strains analyzed.

Studies in the literature further explore (−)-myrtenol, demonstrating its fungicidal activity against strains of *Aspergillus niger* and *Candida* spp., as well as its gastroprotective and healing effects in animal models [22,23,24]. However, investigations regarding the action of (−)-myrtenol on virulence factors expressed by *C. albicans* are still scarce.

Given this context, the present study evaluated the effects of (−)-myrtenol on the virulence factors of *C. albicans* isolated from patients with oral candidiasis, including adhesion to oral epithelial cells, morphogenesis and lipase production.

## 2. Materials and Methods

### 2.1. Study Design

This study is an in vitro experimental laboratory investigation conducted at the Clinical Mycology Laboratory, Department of Pharmaceutical Sciences, Health Sciences Center, Federal University of Paraíba.

Ten strains collected from the oral mucosa of patients with oral candidiasis (Onofre Lopes University Hospital, Rio Grande do Norte, Brazil) and one reference strain (*C. albicans* ATCC 90028) were used in this study. The study was approved by the ethics committee of the Federal University of Rio Grande do Norte (Certificate of Ethical Review Submission—23611519.6.0000.5292) and the strains are stored in the yeast bank of the research laboratory. The collection of epithelial cells was approved by the research ethics committee of the Federal University of Paraíba (Certificate of Ethical Review Submission—28418620.0.0000.5183).

### 2.2. Viability and Samples Purity

The strains were reactivated in liquid Yeast Peptone Dextrose (“YPD”; yeast extract 10 g/L; dextrose 20 g/L; peptone 20 g/L) and incubated at 37 °C for 48 h. Subsequently, they were inoculated onto Sabouraud Dextrose Agar and incubated at 37 °C for 48 h.

To assess purity, yeasts were inoculated into Petri dishes (90 × 15 mm) containing CHROMagar *Candida*^®^ medium (CHROMagarTM *Candida*, Difco, BD, Sparks, MD, USA), a selective chromogenic medium that allows the identification of mixed yeast cultures. The plates were incubated at 37 °C for 48 h, with positivity for *C. albicans* indicated by green-colored colonies. In addition, the strains were previously subjected to microsatellite typing PCR and ABC genotyping, as described by da Silva-Rocha et al. [25].

### 2.3. (−)-Myrtenol Compound

The (−)-myrtenol used in the experiments was purchased from Sigma-Aldrich^®^, São Paulo, SP, Brazil (lot #BCBM4384V).

### 2.4. Minimum Inhibitory Concentration Assay

The minimum inhibitory concentration (MIC) of (−)-myrtenol was determined using the broth microdilution technique in sterile 96-well U-bottom microplates (ALAMAR^®^), according to the Clinical and Laboratory Standards Institute (CLSI) guidelines [26,27], with minor modifications. Yeast suspensions were initially prepared in sterile saline and adjusted to a turbidity equivalent to the 0.5 McFarland standard (approximately 1–5 × 10^6^ CFU/mL). This suspension was subsequently diluted in RPMI 1640 medium (with L-glutamine, without bicarbonate, and buffered with MOPS) to obtain a final inoculum concentration of approximately 0.5–2.5 × 10^3^ CFU/mL in each well. Modifications included the preparation of the inoculum in saline prior to dilution in RPMI 1640 and the use of a defined concentration range of (−)-myrtenol (1024–8 µg/mL).

Serial dilutions of (−)-myrtenol were prepared in RPMI 1640 in U-bottom 96-well microplates, in triplicate, with final concentrations ranging from 1024 to 8 µg/mL. The (−)-myrtenol emulsion was prepared using 5% DMSO and 2% Tween 80. Control assays containing the same concentrations of DMSO (5%) and Tween 80 (2%) were performed to evaluate their potential effects on fungal growth and virulence parameters. No significant interference was observed under these conditions, confirming that the observed effects were attributable to (−)-myrtenol. The plates were incubated at 35 °C for 24 h, and the results were determined by visual observation of fungal growth. The MIC was defined as the lowest concentration of the compound capable of visibly inhibiting fungal growth.

### 2.5. Inoculum Standardization

To phenotypically characterize the isolated *C. albicans* strains, the samples were grown in liquid YPD medium in the presence and absence of (−)-myrtenol (256 μg/mL—Minimum Inhibitory Concentration previously determined). Cells were inoculated by wet looping into the medium (using a loop loaded with a film of yeast suspension, which was quickly immersed in the medium and then removed) and incubated for 18–24 h at 37 °C. This procedure resulted in an inoculum of approximately 2 × 10^8^ cells/mL measured by spectrophotometry at 600 nm optical density (Cary 60 UV-Vis, Agilent Technologies, Santa Clara, CA, USA), with absorbance values ranging from 0.8 to 1.2 [28]. *C. albicans* cells were then diluted to achieve the required inoculum concentration for each virulence attribute assessed. Thus, for comparative purposes, all subsequent experiments were conducted both in the absence and presence of the substance being tested.

### 2.6. Candida albicans Adherence to Human Oral Epithelial Cells

Samples of human oral epithelial cells (HOEC) were collected from healthy volunteers by swabbing the oral mucosa with a sterile swab for 2 min, and then transferred to conical tubes containing 5 mL of PBS (Phosphate-Buffered Saline; NaCl 8 g/L; KCl 0.2 g/L; Na_2_HPO_4_ 1.44 g/L; KH_2_PO_4_ 0.24 g/L, pH 7.2), and kept refrigerated until experimentation. *Candida albicans* cells grown overnight in YPD broth (in the presence and absence of myrtenol) and HOEC were centrifuged at 1200× *g* (4 °C) for 5 min and washed three times with PBS.

The inoculum was standardized to 5 × 10^6^ cells/mL for *C. albicans* and 5 × 10^5^ cells/mL for HOEC. The two cell types were mixed in equal proportions (100 µL of each suspension), in triplicate, and then incubated at 37 °C for 1 h. Following the 1 h co-incubation, the cells were fixed in formalin. The total number of adherent *Candida albicans* cells was determined by manually counting the yeast cells adhered to 150 human oral epithelial cells (HOEC) under a light microscope (40× objective). A standardized and systematic microscopic scanning method was employed, with fields examined sequentially from bottom to top and from left to right, ensuring that observed areas were not overlapped. All samples were analyzed in triplicate using consistent counting criteria. This approach is widely used in adhesion assays involving *Candida* spp. and epithelial cells [29,30].

### 2.7. Morphogenesis Assay

The morphogenesis assay was conducted based on the technique described by Chaves et al. [28] with the following modifications: standardization of the inoculum to 1 × 10^6^ cells/mL, use of YPD supplemented with 20% fetal bovine serum as an inducer, and fixation of aliquots with 10% formalin at defined time points (1 h and 3 h) for subsequent microscopic analysis. The *C. albicans* cells were grown overnight (18–24 h) in YPD medium in the presence and absence of (−)-myrtenol. The absorbance of cellular growth was measured using a spectrophotometer (Cary 60 UV-Vis, Agilent Technologies, USA) at 600 nm, and the concentration was standardized to 1 × 10^6^ cells/mL.

To induce morphogenesis, 30 µL of the standardized inoculum was added to YPD broth supplemented with 20% fetal bovine serum and incubated at 37 °C. After 1 h of incubation, a 500 µL aliquot was removed and immediately fixed with 10% formalin for germ tube evaluation. The remaining culture was returned to incubation for an additional 2 h, completing a total of 3 h. After this period, a second 500 µL aliquot was collected and fixed with 10% formalin for subsequent morphological index analysis. Fixed samples were stored at 4 °C until microscopic examination.

All slides were read in triplicate. The slides from the 1 h incubation were evaluated by counting 100 cells per slide and determining the percentage of cells that exhibited germ tube formation.

Regarding the slides from the samples subjected to 3 h of incubation, the Morphology Index was calculated, as greater morphological variability of *C. albicans* cells is expected. Therefore, 100 *C. albicans* cells were counted on each slide, with the following morphology classifications: blastoconidia (round cells) were assigned an MI value of 1; elongated cells with a diameter twice the length were assigned MI = 2; cells resembling pseudohyphae were assigned MI = 3; and long, true hyphae with parallel sides were assigned MI = 4 [31]. The Morphology Index (MI) was determined using the following formula:

$$Morphology\;Index\left(MI\right)=\frac{\left(N{}^{\circ}\;MI1\;\times \;1)\;+\;(N{}^{\circ}\;MI2\;\times \;2)\;+\;(N{}^{\circ}\;MI3\;\times \;3)\;+\;(N{}^{\circ}\;MI4\;\times \;4\right)}{100}$$

In this context, values close to one indicate a population of spherical yeast cells; values close to four indicate a population of true hyphae; and values between one and four suggest the presence of varied morphologies or, predominantly, pseudohyphae.

### 2.8. Measurement of Candida albicans Hyphal Length

The cell length of *C. albicans*, specifically strain 97, obtained from independent experiments, was measured after the induction of morphogenesis (incubation for 3 h in YPD + 20% FBS) [32]. The NIS-Elements D software (version 6.20.00) was used for this purpose. For each strain, the average length of 100 hyphal cells was determined for isolates previously cultured in the presence or absence of (−)-myrtenol.

### 2.9. Morphogenesis of Candida albicans on Solid Media

To induce hyphal formation on solid medium, cells were cultured in YPD. A 10 μL aliquot of the standardized cell suspensions was inoculated onto the surface of Spider medium (nutrient agar 10 g, mannitol 10 g, KH_2_PO_4_ 2 g, agar 14.5 g, distilled water 1000 mL) in the presence and absence of 256 µg/mL of (−)-myrtenol. The compound was added to the culture medium after autoclaving, at the same concentration used in liquid assays, ensuring continuous exposure of the strains throughout the incubation period [33]. The plates were incubated at 37 °C for seven days in order to observe the macromorphological aspects of the colonies. The assay was performed in triplicate [31].

### 2.10. Lipase Enzyme Production

Lipase Index (LI) was assessed using the methodology proposed by Muhsin et al. [34] with the following modifications: the use of Tween 80 as a substrate, standardization of the inoculum to 2 × 10^5^ cells/mL, and maintenance of (−)-myrtenol in the culture medium throughout the incubation period. Initially, cultures grown overnight in YPD broth with and without (−)-myrtenol were diluted and standardized to a concentration of 2 × 10^5^ cells/mL. (−)-Myrtenol was added to the culture medium after autoclaving, at the same concentration used in liquid assays, ensuring continuous exposure of the strains throughout the incubation period.

Subsequently, 10 µL of each strain suspension was cultivated on sterile Petri dishes containing lipid medium (peptone 1%, sodium chloride 5%, calcium chloride 0.01%, and agar 2%, plus 1% Tween 80) and incubated at 37 °C for 5 days. After the incubation period, the diameters of the colonies and the halos formed were measured. LI was determined using the following equation:

$$LI=\frac{Colony\;Diameter\;(cm)}{Colony\;Diameter\;\left(cm\right)\;+Precipitation\;Zone\;(cm)}\;$$

An LI of 0–0.33 was considered strong, an LI of 0.34–0.66 was considered moderate, an LI of 0.67–0.99 was considered weak, and an LI of 1 indicated no lipase activity.

### 2.11. Statistical Analysis

Data were analyzed using the statistical software GraphPad Prism, version 3.0. Results were expressed as mean ± standard deviation. For comparisons between the same strains under different conditions (absence and presence of (−)-myrtenol), the paired Student’s *t*-test was applied. When the assumption of normality was not met, the Wilcoxon signed-rank test was used as a non-parametric alternative. A *p*-value < 0.05 and a 95% confidence interval were considered statistically significant.

## 3. Results

### 3.1. Minimum Inhibitory Concentration Assay

All strains exhibited a MIC of 256 µg/mL, which was used in subsequent assays.

### 3.2. Candida albicans Adherence to Human Oral Epithelial Cells

To evaluate the adhesion capacity of *Candida albicans*, yeast cells were co-incubated with HOEC, and the number of yeast cells adhered to HOEC was assessed by light microscopy. In general, (−)-myrtenol reduced the adhesion capacity in all *C. albicans* strains tested. In the absence of (−)-myrtenol, the mean adhesion was 92.24/150 HOEC, while in the presence of (−)-myrtenol, the mean adhesion was reduced to 28.69/150 HOEC.

**Figure 1:**
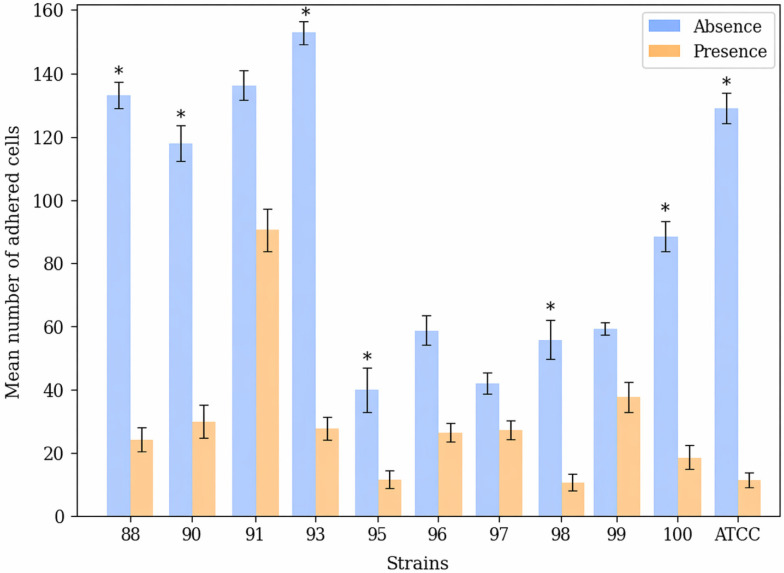
*Candida albicans* adherence to human oral epithelial cells. Mean number of *Candida albicans* yeast cells adhering to 150 human oral epithelial cells, in the presence and absence of 256 µg/mL (−)-myrtenol. The error bars represent the standard deviation. * *p* < 0.05.

**Figure 2:**
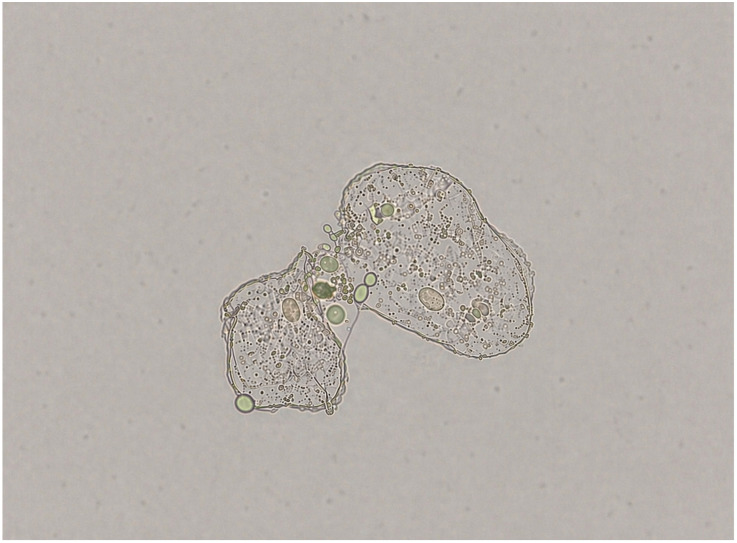
Assay of adhesion to human oral epithelial cells. Epithelial cell showing adhered *Candida albicans* cells. Observations were performed using light microscopy at 40× magnification.

Strain 93 showed the highest adhesion to HOEC (154.00 ± 38.97) and the lowest response when grown in the presence of (−)-myrtenol (27.33 ± 9.29). Strain 95 showed the lowest adhesion in the absence of (−)-myrtenol, but even this reduced property was further affected in the presence of the compound (39.33 ± 5.13 vs. 12.33 ± 3.51). Data from all clinical isolates evaluated in the adhesion assay are presented in Table 1 and Figure 1. Figure 2 illustrates the adhesion assay.

### 3.3. Morphogenesis Assay

**Figure 3:**
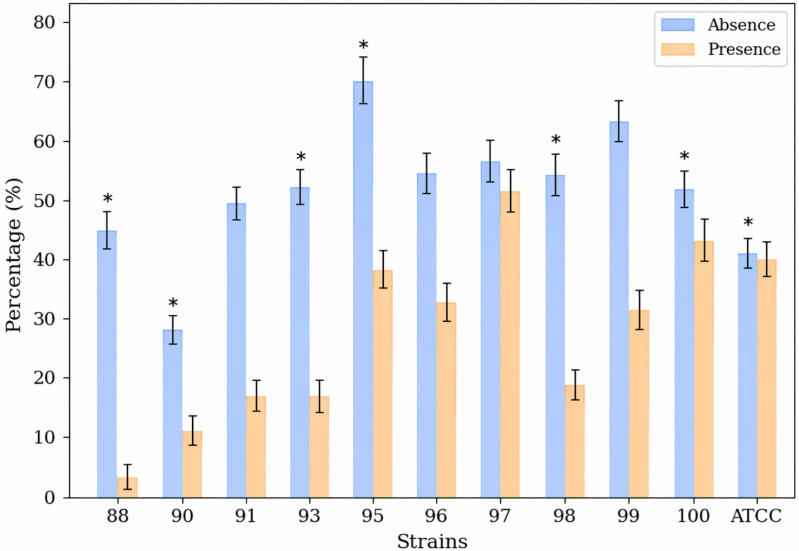
Morphogenesis assay (1 h). Mean percentages of germ tube formation in liquid medium (YPD + 20% FBS) after 1 h of incubation at 37 °C in the absence and presence of 256 µg/mL (−)-myrtenol. The error bars represent the standard deviation. * *p* < 0.05.

The effect of (−)-myrtenol on *C. albicans* filamentation was evaluated using serum and a temperature of 37 °C as inducing agents. After 1 h of incubation, the germ tube formation capacity was evaluated. The overall mean percentage of germ tube formation for the strains evaluated was 51.7% in the absence of (−)-myrtenol. A reduction was observed in the presence of the compound, with a mean formation rate of 27.8%. Strain 88 showed the greatest reduction (44.7% vs. 3%), as described in Figure 3.

After 3 h of incubation, the Morphology Index was calculated, since different morphological aspects can be observed after this period. It was observed that in the absence of (−)-myrtenol, pseudohyphae predominated, with a mean MI of 3.17 among the strains evaluated. In the presence of the compound, this decreased to a MI of 2.57.

**Figure 4:**
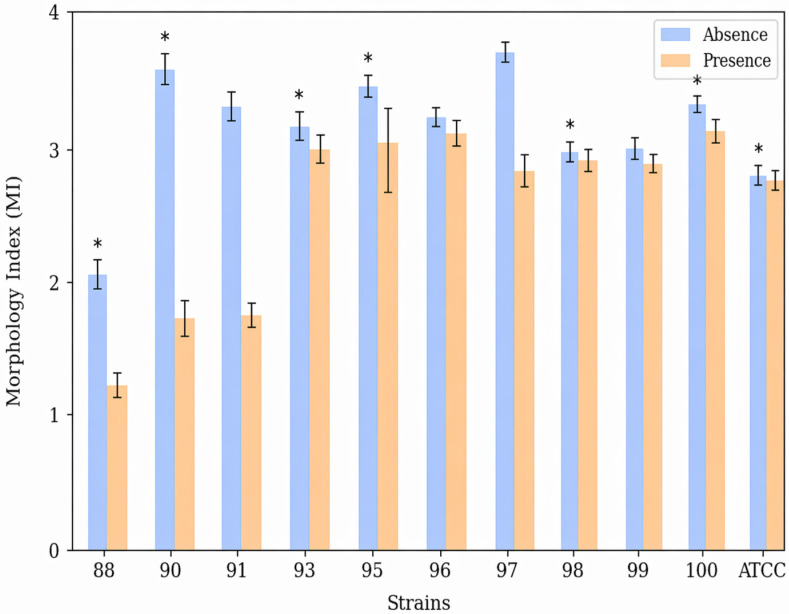
Morphogenesis assay (3 h). Mean Morphology Index (MI) in liquid medium (YPD + 20% FBS) after 3 h of incubation at 37 °C in the absence and presence of 256 µg/mL (−)-myrtenol. The error bars represent the standard deviation. * *p* < 0.05.

**Figure 5:**
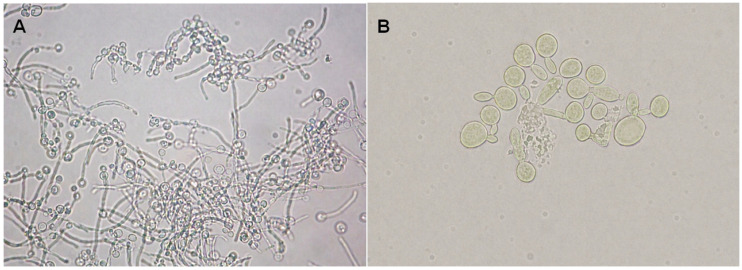
Morphogenesis in *Candida albicans* in YPD Broth supplemented with 20% Fetal Bovine Serum (37 °C). (**A**) Strain 97 illustrating the dense formation of elongated, true hyphae in the absence of (−)-myrtenol. (**B**) Microscopic representation of the assay conducted in the presence of (−)-myrtenol, demonstrating a reduced capacity for morphogenesis, characterized by the presence of few and stunted germ tubes (40× magnification).

Strain 97 was highly filamentous, with a MI of 3.84 predominantly forming long true hyphae. In the presence of (−)-myrtenol, the Morphology Index was reduced to 2.84. Strain 90 showed the best result in reducing hyphae formation, with a MI of 3.64 in the absence of (−)-myrtenol and a MI of 1.73 in the presence of the compound (Figure 4). Figure 5 illustrates the formation of true hyphae when myrtenol is absent, and the inhibited formation of germ tubes when myrtenol is present.

### 3.4. Measurement of Candida albicans Hyphal Length

After evaluating the results obtained from the morphogenesis phase in liquid medium (3 h), it was found that strain 97 had a greater filamentation capacity than the other strains. In the presence of the substance, a reduction in hyphal length was observed in strain 97, which was selected due to its high filamentation capacity. Strain 97 was selected for measurement using an optical microscope and NIS-Elements D software (Eclipse Ci, Nikon Corporation, Tokyo, Japan). In the absence of (−)-myrtenol, most of the analyzed hyphae had an overall average size of 92.09 micrometers (92.09 ± 21.34), which decreased to 77.42 micrometers (77.42 ± 17.56) in the presence of (−)-myrtenol, representing a statistically significant reduction. The results are presented as mean ± standard deviation.

### 3.5. Morphogenesis of Candida albicans on Solid Media

**Figure 6:**
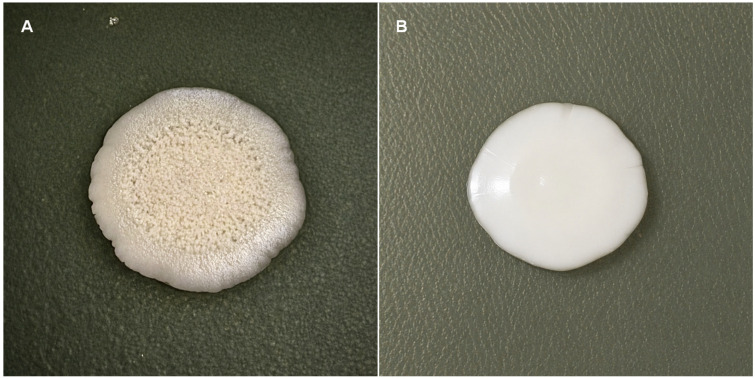
Morphogenesis in *C. albicans* observed by induction in solid medium using Spider agar for 7 days. (**A**) Strain 97 exhibiting a filamentous phenotype when cultured in the medium without (−)-myrtenol. (**B**) Spider agar with 256 µg/mL of (−)-myrtenol, with colony presenting a smooth phenotype.

Morphogenesis in *C. albicans* was also observed by induction on solid medium using Spider medium for 7 days. Strains 97 and 99 exhibited a filamentous phenotype when grown on the medium without (−)-myrtenol. When inoculated on Spider agar with (−)-myrtenol, the colonies showed a smooth phenotype (Figure 6). It was observed that the macroscopic appearance of the colonies for most isolates remained smooth both in the presence and absence of the substance (Table 2).

### 3.6. Lipase Enzyme Production

The effect of (−)-myrtenol on lipase enzyme secretion was investigated in the 11 study strains. After the incubation period, the diameters of the colonies and halos formed were measured, and the Lipase Index (LI) was calculated. In the absence of (−)-myrtenol, most isolates exhibited lipase activity, with a mean LI of 0.68, indicating weak enzymatic activity. However, strains 98 and 100 showed an LI of 1, corresponding to absence of lipase production under baseline conditions. In the presence of (−)-myrtenol, inhibition of lipase activity was observed in all strains that exhibited baseline enzyme production, as indicated by LI values equal to 1. These findings indicate that (−)-myrtenol suppresses lipase production in lipase-producing strains. Table 3 presents the means as well as the standard deviations for each strain analyzed.

## 4. Discussion

The present study aimed to evaluate the in vitro action of (−)-myrtenol, a compound with documented antifungal activity, on various virulence factors expressed by *Candida albicans* [23,35]. To our knowledge, this is the first study to address the effect of this monoterpene on the following virulence factors: adhesion, morphogenesis and hydrolytic lipase enzyme activity.

Myrtenol is a monoterpene primarily found in a plant commonly known as myrtle (*Myrtus communis*). This plant belongs to the Myrtaceae family and possesses antimicrobial properties (antibacterial, antifungal, and antiviral), as well as cardioprotective, antioxidant, and anti-inflammatory properties, among others [36]. Research has already reported that the enantiomer as (−)-myrtenol exhibits antifungal activity and reduces biofilm formation when associated with other medications such as antibiotics and antifungals [21,23]. In addition to *M. communis*, other species within the Myrtaceae family, such as *Eugenia uniflora* (1000 μg/mL) and *Leptospermum scoparium* (20 μg/mL), also exhibit described antifungal activities [29,37].

Regarding the influence of (−)-myrtenol on *Candida albicans* adhesion, a significant reduction in this attribute was observed in all strains analyzed. To date, this finding is unprecedented, since there are no reports in the literature on the evaluation of (−)-myrtenol on the adhesion of *C. albicans* to epithelial cells. Souza et al. [29] studied the effect of *Eugenia uniflora* leaf extract on *Candida* virulence factors and, using a similar methodology, observed a significant reduction in adhesion.

It is known that microbial adhesion to epithelial cells is the first step towards colonization and, subsequently, towards the establishment of an infectious process. The action of (−)-myrtenol, which promotes the reduction in adhesion, may contribute to reducing the establishment of infection. Furthermore, adhesin proteins, responsible for cell recognition and adhesion, are anchored in the cell wall, suggesting that this may be a potential target for (−)-myrtenol. One limitation of this experiment is the precision of quantifying *Candida albicans* adhesion to epithelial cells, as the counting process depends on the observer and may be subject to observer-dependent variability.

When analyzing the morphogenetic process of *Candida albicans*, the results of this study indicate that (−)-myrtenol exerts an inhibitory effect on the phenotypic transition of the strains evaluated. Morphological changes that culminate in the formation of true hyphae are a key factor in virulence, leading to a greater ability to cause infections. Notably, a reduction in germ tube formation was observed in the evaluated strains when (−)-myrtenol was applied. The first hour of filamentation induction is the period during which germ tubes are formed, and the action of (−)-myrtenol in delaying this process may reflect a reduced ability of the yeast to progress in the infection process.

After 3 h of filamentation induction, the formation of true hyphae is observed. In this study, a reduction in the formation of these structures and a predominance of short pseudohyphae were noted. The clinical isolate 97 used in the study is a strain with a high capacity for hyphae formation (MI 3.84). However, under the action of (−)-myrtenol, the MI was reduced to 2.84. For this same strain, there was a decrease in length when the compound was present. It is important to note that hyphal length measurements were performed only for strain 97, selected due to its high filamentation capacity. Therefore, this observation should be interpreted as representative of this specific strain and not generalized across all isolates. This finding reinforces that even in situations of complex morphology, (−)-myrtenol was able to reduce filamentation.

In a study carried out by Silva-Rocha et al., the action of the crude extract of *Eugenia uniflora*, a terpene, on morphogenesis was evaluated under different induction conditions similar to this study [32]. They observed that the predominant morphology was that of pseudohyphae, with reduced filamentation after the use of the extract. These results are in line with the data presented in the current study, indicating that (−)-myrtenol acts by delaying or inhibiting the filamentation of *Candida albicans*. The same authors also pointed out a reduction in the length of the hyphae evaluated as well as in the filamentation of colonies on solid medium.

In the morphogenesis assay on solid medium (Spider agar), it was observed that most isolates maintained a smooth colony phenotype regardless of the presence of (−)-myrtenol. Although Spider medium is widely recognized as a strong inducer of filamentation in *Candida albicans*, variability in morphogenetic potential among clinical isolates is well documented. Differences in filamentation capacity may be associated with strain-specific regulatory pathways and environmental responsiveness [12,13]. In this context, only strains 97 and 99 exhibited a filamentous phenotype under control conditions, which was reduced in the presence of (−)-myrtenol. These findings indicate that the effect of the compound is more evident in strains with an intrinsic capacity for filamentation, whereas isolates with limited morphogenetic potential may not display phenotypic changes under the same conditions.

Recent studies have evaluated the use of other terpenes such as carvacrol, limonene, thymol, eugenol, and observed that these compounds were able to reduce *Candida albicans* filamentation at different concentrations [38,39]. This is the first study to report the action of (−)-myrtenol on this specific virulence factor.

Our findings suggest that (−)-myrtenol has potential as a therapeutic agent in candidiasis. The process of morphogenesis significantly contributes to tissue invasion, as the extension of hyphae facilitates tissue erosion and consequently, greater damage to the host. Therefore, by affecting the formation of filamentous structures, (−)-myrtenol may impede the progression of tissue damage during infection [40]

In a study by Silva-Rocha et al. [41] proteomic analysis was used to identify proteins affected during morphogenesis by the ethyl acetate fraction of the crude extract of *Eugenia uniflora*. The findings showed that cell wall proteins directly involved in hyphal elongation had reduced expression. The findings of the present study suggest that similar mechanisms may be involved, possibly affecting cell wall-associated proteins.

Several species of the *Candida* genus, such as *C. rugosa*, *C. guilliermondii*, *C. parapsilosis*, *C. tropicalis*, and *C. albicans* secrete lipase as a virulence factor [42,43]. This enzyme, due to its ability to lyse lipids, facilitates invasion and colonization of host tissues [44,45]. The inhibitory effect of (−)-myrtenol on lipase production was evident in strains that exhibited basal enzymatic activity. It is important to note that some isolates (e.g., strains 98 and 100) did not produce lipase under control conditions, highlighting intrinsic variability among clinical isolates. Therefore, the observed effect of (−)-myrtenol is restricted to lipase-producing strains.

A study conducted by Prasath et al. [46] using palmitic acid to inhibit virulence factors in *Candida tropicalis* reported a significant reduction in lipase production at a concentration of 200 μg/mL and 400 μg/mL after 48 h of incubation. In *C. albicans*, ten lipases (Lip1-10) have been described, each acting at different stages of the infectious process. Among these, Gácser et al. [47] demonstrated that mutant strains of lipase 8 exhibited reduced virulence in a murine infection model. Therefore, the action of (−)-myrtenol in preventing the secretion of lipases in *C. albicans* may also indicate an important interaction in reducing the virulence of this pathogen.

The observed effects of (−)-myrtenol on adhesion, morphogenesis, and lipase production suggest interference with key virulence attributes of *Candida albicans*. However, as the experiments were conducted at the minimum inhibitory concentration (MIC), these findings should be interpreted as modulation of virulence-associated phenotypes under MIC-level exposure, which may also reflect the effects of growth inhibition or cellular stress. Although possible mechanisms may involve modulation of cell wall components or adhesin-related pathways, these interpretations remain speculative and were not directly assessed in the present study. Further studies using sub-inhibitory concentrations would help to refine the understanding of these effects.

## 5. Conclusions

This study demonstrates that (−)-myrtenol significantly modulates key virulence factors of *Candida albicans*, including adhesion to epithelial cells, morphogenesis, and lipase production. Notably, this is one of the first studies to evaluate the effects of (−)-myrtenol on these virulence attributes in clinical isolates from the oral cavity. The compound reduced adhesion capacity, impaired filamentation, and inhibited lipase activity in strains exhibiting basal enzyme production, highlighting its impact on different stages of fungal pathogenicity. These findings contribute to the understanding of the anti-virulence potential of natural compounds and support (−)-myrtenol as a promising candidate for further investigation. However, additional studies are required to elucidate its mechanisms of action and to assess its applicability in clinical settings.

## Figures and Tables

**Table 1:** Adherence of *Candida albicans* to human oral epithelial cells in the absence and presence of (−)-myrtenol.

Strain	Absence of Myrtenol (Mean ± SD)	Presence of Myrtenol (Mean ± SD)	% Reduction	*p*-Value
**Strain 88**	134.7 ± 23.6	23.0 ± 11.0	82.9%	0.018
**Strain 90**	117.7 ± 5.5	29.0 ± 8.9	75.4%	0.002
**Strain 91**	137.3 ± 6.0	90.3 ± 18.8	34.2%	0.050
**Strain 93**	154.0 ± 39.0	27.3 ± 9.3	82.3%	0.023
**Strain 95**	39.3 ± 5.1	12.3 ± 3.5	68.7%	0.027
**Strain 96**	58.7 ± 15.0	26.7 ± 5.5	54.5%	0.110
**Strain 97**	41.7 ± 10.5	28.0 ± 1.0	32.9%	0.165
**Strain 98**	55.0 ± 5.6	10.7 ± 2.3	80.6%	0.010
**Strain 99**	59.0 ± 15.0	37.7 ± 11.6	36.1%	0.196
**Strain 100**	88.0 ± 9.0	18.0 ± 8.0	79.5%	0.014
**ATCC 90028**	129.3 ± 10.7	12.7 ± 1.5	90.2%	0.003

Data are expressed as mean ± standard deviation (SD) of triplicate experiments. Statistical analysis was performed using the paired Student’s *t*-test.

**Table 2:** Macroscopic colony morphology of *Candida albicans* isolates on Spider medium in the absence and presence of (−)-myrtenol. The minimum inhibitory concentration (MIC) of (−)-myrtenol was 256 µg/mL.

Spider Medium	
Strain	Absence vs. Presence of (−)-Myrtenol
Strain 88	Smooth vs. Smooth
Strain 90	Smooth vs. Smooth
Strain 91	Smooth vs. Smooth
Strain 93	Smooth vs. Smooth
Strain 95	Smooth vs. Smooth
Strain 96	Smooth vs. Smooth
Strain 97	Wrinkled vs. Smooth
Strain 98	Smooth vs. Smooth
Strain 99	Wrinkled vs. Smooth
Strain 100	Wrinkled vs. Wrinkled
ATCC 90028	Smooth vs. Smooth

**Table 3:** Lipase production index. Mean lipase activity in *Candida albicans* isolates in the presence and absence of (−)-myrtenol. * *p* < 0.05. The minimum inhibitory concentration (MIC) of (−)-myrtenol was 256 µg/mL.

Lipase Index (LI)		
*Candida albicans*	Absence of (−)-Myrtenol	Presence of (−)-Myrtenol
Strain 88	0.53 ± 0.05	1.0 ± 0.0 *
Strain 90	0.65 ± 0.03	1.0 ± 0.0 *
Strain 91	0.55 ± 0.11	1.0 ± 0.0 *
Strain 93	0.61 ± 0.05	1.0 ± 0.0 *
Strain 95	0.46 ± 0.06	1.0 ± 0.0 *
Strain 96	0.97 ± 0.06	1.0 ± 0.0 *
Strain 97	0.55 ± 0.05	1.0 ± 0.0 *
Strain 98	1.0 ± 0.0	1.0 ± 0.0 *
Strain 99	0.55 ± 0.04	1.0 ± 0.0 *
Strain 100	1.0 ± 0.0	1.0 ± 0.0 *
Strain ATCC 90028	0.65 ± 0.16	1.0 ± 0.0 *

## Data Availability

The datasets used and/or analysed during the current study are available from the corresponding author on reasonable request.

## Abbreviations

The following abbreviations are used in this manuscript:

YPD	Yeast Peptone Dextrose
MIC	Minimum Inhibitory Concentration
HOEC	Human Oral Epithelial Cells
FBS	Fetal Bovine Serum
MI	Morphology Index
LI	Lipase Index
